# Histone H2AFX Links Meiotic Chromosome Asynapsis to Prophase I Oocyte Loss in Mammals

**DOI:** 10.1371/journal.pgen.1005462

**Published:** 2015-10-28

**Authors:** Jeffrey M. Cloutier, Shantha K. Mahadevaiah, Elias ElInati, André Nussenzweig, Attila Tóth, James M. A. Turner

**Affiliations:** 1 The Francis Crick Institute, Mill Hill Laboratory, London, United Kingdom; 2 Laboratory of Genome Integrity, National Cancer Institute, NIH, Bethesda, Maryland, United States of America; 3 Institute of Physiological Chemistry, Technische Universität Dresden, Dresden, Germany; Cornell University, UNITED STATES

## Abstract

Chromosome abnormalities are common in the human population, causing germ cell loss at meiotic prophase I and infertility. The mechanisms driving this loss are unknown, but persistent meiotic DNA damage and asynapsis may be triggers. Here we investigate the contribution of these lesions to oocyte elimination in mice with chromosome abnormalities, e.g. Turner syndrome (XO) and translocations. We show that asynapsed chromosomes trigger oocyte elimination at diplonema, which is linked to the presence of phosphorylated H2AFX (γH2AFX). We find that DNA double-strand break (DSB) foci disappear on asynapsed chromosomes during pachynema, excluding persistent DNA damage as a likely cause, and demonstrating the existence in mammalian oocytes of a repair pathway for asynapsis-associated DNA DSBs. Importantly, deletion or point mutation of *H2afx* restores oocyte numbers in XO females to wild type (XX) levels. Unexpectedly, we find that asynapsed supernumerary chromosomes do not elicit prophase I loss, despite being enriched for γH2AFX and other checkpoint proteins. These results suggest that oocyte loss cannot be explained simply by asynapsis checkpoint models, but is related to the gene content of asynapsed chromosomes. A similar mechanistic basis for oocyte loss may operate in humans with chromosome abnormalities.

## Author Summary

Chromosome abnormalities, such as aneuploidies and structural variants (i.e. translocations, inversions), are strikingly common in the human population, causing disorders such as Down syndrome and Turner syndrome. One important consequence of chromosome abnormalities in mammals is errors during meiosis, the specialized cell division that generates sperm and eggs for reproduction. As a result of these meiotic errors, patients with chromosome abnormalities oftentimes suffer from infertility due to loss of developing germ cells. The precise molecular mechanism for germ cell losses and infertility due to chromosome abnormalities is not well understood, but is hypothesized to result from a surveillance mechanism, which has evolved to prevent aneuploidies from developing from abnormal germ cells. In mammals, meiotic surveillance mechanisms have been hypothesized to monitor for unrepaired DNA double-strand breaks (DSB) and/or chromosome pairing/synapsis errors. Here we test these hypotheses using a variety of chromosomally variant mouse models. We find that germ cell loss in female mice with chromosome abnormalities is dependent on phosphorylation of the histone variant H2AFX, an epigenetic mark involved in the transcriptional silencing of asynapsed chromosomes during meiosis. These data inform a silencing-based mechanism of germ cell loss in patients with chromosome abnormalities and for the prophase I surveillance system which safeguards against aneuploidy.

## Introduction

Prophase I of mammalian meiosis entails alignment, synapsis and reciprocal recombination between homologues, which together enable crossover formation. Without crossovers, homologues mis-segregate, giving rise to aneuploidy [1]. To protect against this, germ cells exhibiting defects in the key prophase I events are eliminated by quality control mechanisms [2]. Understanding the molecular basis of these surveillance mechanisms represents an important challenge.

The pathways that drive prophase I oocyte loss in chromosomally abnormal female mice are unclear. Two models have so far predominated: persistent meiotic DNA damage and chromosome asynapsis. Evidence that persistent DNA damage can cause oocyte loss is derived from studies of mice carrying targeted mutations in meiotic recombination genes, e.g. *Dmc1* [3]. In these models, DNA DSB markers, e.g. RAD51 and DMC1, persist at chromosome axes [4] and oocyte loss is partially rescued by ablating *Spo11* [3], the enzyme responsible for meiotic DNA DSB formation. Persistent DNA damage triggers oocyte elimination via the CHK2/p53/p63 pathway [5]. It is important to note that in these targeted mutants DNA repair pathways are genetically disabled, while in chromosomally abnormal mice they are intact. Whether meiotic DNA DSBs persist in chromosomally abnormal mice therefore remains an open question.

Mouse mutants in which chromosome asynapsis is present but programmed meiotic DNA DSB formation is either reduced or abolished e.g. *Mei1-/-* [6], *Mei4-/-* [7] and *Spo11 -/-* [3,8,9] also exhibit prophase I elimination. This suggests that asynapsis *per se* can also cause oocyte loss. Asynapsis has been observed in chromosomally abnormal female mice [10,11], and leads to accumulation of HORMAD1/2 at chromosome axes [12–14] and serine-139 phosphorylated histone H2AFX (γH2AFX) in the adjacent chromatin [15,16]. How these changes precipitate oocyte elimination is not known. One hypothesis invokes the existence of a synapsis checkpoint that eliminates oocytes in response to asynapsis *per se* [17]. An alternative model suggests a role for γH2AFX-associated transcriptional inactivation [2,18]. This process, known as meiotic silencing, could cause elimination through silencing of essential germ cell-expressed genes, or through alterations in transcription factor binding on asynapsed chromosomes, or expression of non-coding RNAs or transposable elements. The synapsis checkpoint and meiotic silencing models are difficult to dissect experimentally, however, because genes with putative synapsis checkpoint functions, e.g. *Hormad1* and *Hormad2*, are also essential for silencing [13,19–21]. Discriminating between these models requires analysis of mice carrying extra/supernumerary chromosomes, in which asynapsis is present but silencing affects genes that are not essential for germ cell development.

Further complexity in understanding the prophase I surveillance mechanisms comes from studies in males. During normal male meiosis, the X and Y chromosomes are asynapsed and thus undergo meiotic silencing. This process, called Meiotic Sex Chromosome Inactivation (MSCI) [22], is highly conserved in eutherian mammals. The fact that the X and Y chromosomes are enriched in HORMAD1, HORMAD 2, γH2AFX and other checkpoint factors, yet do not trigger prophase I elimination, is puzzling.

Here we examine the pathways that drive oocyte loss in an extensive array of mice carrying chromosome abnormalities similar to those found in humans. We present a model for prophase I elimination that unifies existing data and explains why in males the asynapsed X and Y chromosomes do not trigger loss while asynapsed autosomes do.

## Results

### Oocytes with asynapsed chromosomes are eliminated during diplonema

Initially, we sought to establish the timing of oocyte loss in mice carrying chromosome abnormalities. We first studied females with sex chromosome abnormalities, using X chromosome monosomy (XO; Turner syndrome) as our model. XO mice have a shortened reproductive lifespan owing to perinatal oocyte losses that have been hypothesized to result from X chromosome asynapsis during prophase I [23,24]. To determine the developmental timing of XO oocyte losses, we examined the percentage of oocytes with X chromosome asynapsis at successive meiotic prophase I substages. We utilized an immunostaining approach to categorize XO oocytes as being in pachynema, when autosomal synapsis is complete, or early and late diplonema, when chromosomes progressively desynapse. Our substaging criteria were based on the immunostaining patterns of SYCP3, an axial element marker, and HORMAD1, a marker of asynapsed and desynapsed axes (S1A–S1C Fig). To identify the asynapsed X chromosome, we simultaneously immunostained for serine-139 phosphorylated H2AFX (γH2AFX), which marks the chromatin of asynapsed chromosomes, but not desynapsed chromosomes, from pachynema to diplonema [16,25].

**Fig 1:**
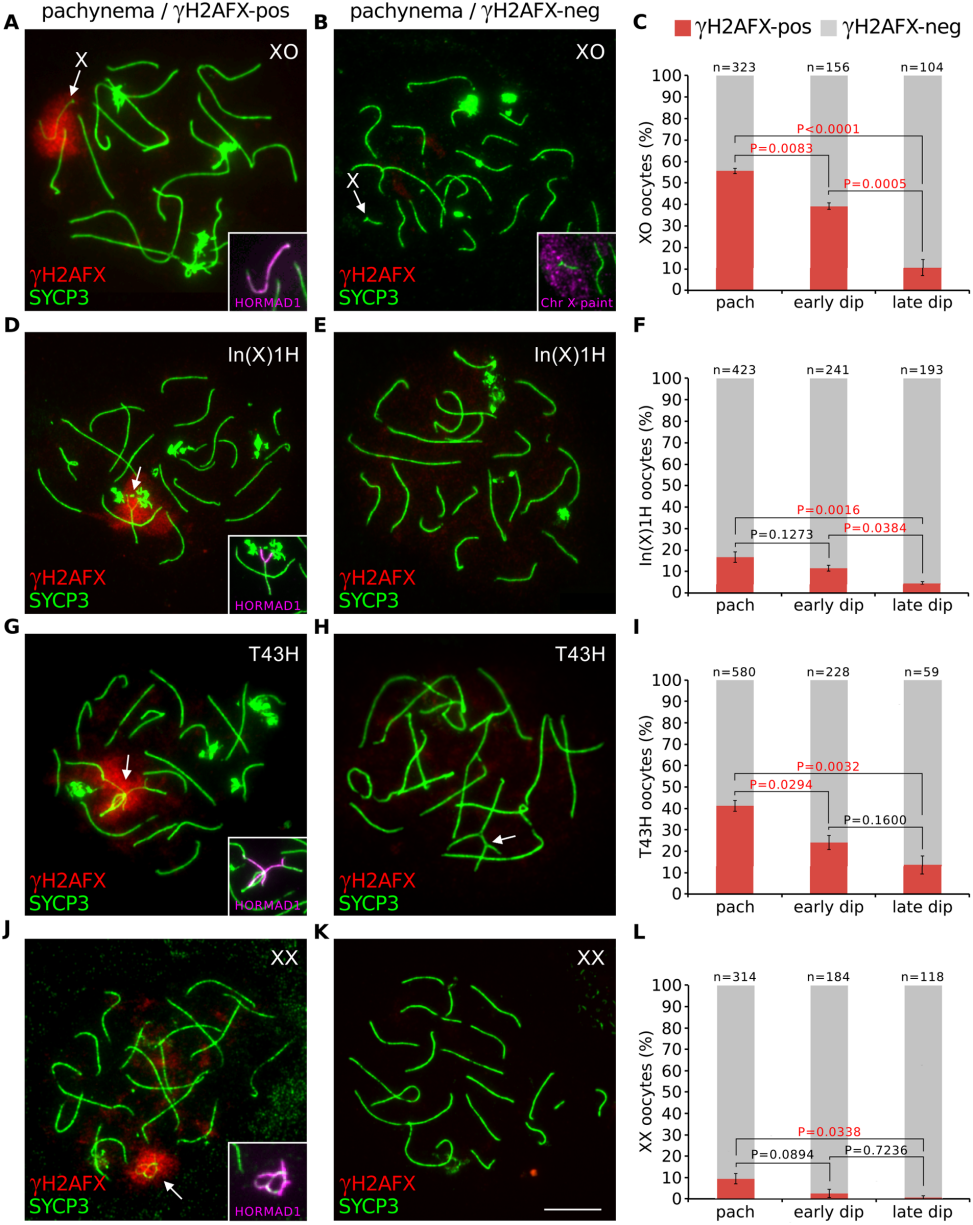
Oocytes with asynapsed chromosomes are eliminated during diplonema. (**A**) XO pachytene oocyte with asynapsed X chromosome (arrow). SYCP3 (green) marks chromosome axes, γH2AFX (red) marks chromatin associated with asynapsed axes, and HORMAD1 (magenta, insets) marks asynapsed axes. (**B**) XO pachytene oocyte with self-synapsed X chromosome. The self-synapsed X chromosome (arrow) was identified by X chromosome painting (magenta, inset). (**C**) The mean percentage (± s.e.m.) of XO oocytes with a γH2AFX-positive or γH2AFX-negative X chromosome at pachynema, early diplonema and late diplonema. (**D**) In(X)1H pachytene oocyte with asynapsis (arrow). (**E**) In(X)1H pachytene oocyte with fully synapsed chromosomes. (**F**) Mean percentage of In(X)1H oocytes with γH2AFX-positive or γH2AFX-negative X chromosomes at pachynema, early diplonema and late diplonema. (**G**) T43H pachytene oocyte with asynapsed autosomes (arrow). (**H**) T43H pachytene oocyte with fully synapsed chromosomes, showing a trivalent involving the translocated chromosomes (arrow). (**I**) Mean percentage of T43H oocytes with γH2AFX-positive or γH2AFX-negative autosomes at pachynema, early diplonema and late diplonema. (**J**) XX pachytene oocyte with asynapsis (arrow). (**K**) XX pachytene oocyte with fully synapsed chromosomes. (**L**) Mean percentage of XX oocytes with γH2AFX-positive or γH2AFX-negative chromosomes at pachynema, early diplonema and late diplonema. P values were calculated from Tukey multiple comparison tests. Significant P values (P<0.05) are denoted by red font. Scale bar represents 10μm. (M) Schematic illustrating the possible outcomes for oocytes with chromosome abnormalities.

At pachynema, we found that 55% of XO oocytes carried an asynapsed, γH2AFX-enriched X chromosome (Fig 1A and 1C). In the remaining oocytes, the X chromosome was self-synapsed and, as previously reported [16], was devoid of γH2AFX (Fig 1B and 1C). Importantly, oocytes with an asynapsed X chromosome decreased in abundance by more than five-fold during diplotene progression (Fig 1C). We confirmed this result using a second approach, in which we quantified the percentage of all XO oocytes with a γH2AFX-positive X chromosome at 17.5, 18.5 and 19.5 days *post-coitum* (d*pc*; S2A and S2B Fig), the developmental time period when oocytes progress semi-synchronously from pachynema to diplonema.

Next, we examined whether the drop in the percentage of XO oocytes with an asynapsed X chromosome was also evident earlier, specifically during pachynema. To test this, we compared the percentage of oocytes with γH2AFX domains at early pachynema and late pachynema. We categorized oocytes into pachytene substages using a modification of a previously described method based on immunostaining for the DNA DSB marker RPA [26]. Foci of RPA are present on synapsed autosomes at early pachynema but disappear thereafter, with few left by late pachynema. Pachytene oocytes were subdivided into those with >30 autosomal RPA foci (early pachynema) and those with ≤30 autosomal RPA foci (late pachynema). No difference in the percentage of oocytes with a γH2AFX-enriched X chromosome was observed between these two populations (S2C Fig). Thus the decrease does not begin until diplonema. The drop in the percentage of XO oocytes with an asynapsed X chromosome during diplonema cannot be explained by conversion of asynapsed X chromosomes to a synapsed configuration, because chromosomes desynapse during this stage of prophase I. We therefore conclude that oocytes with an asynapsed X chromosome are eliminated during diplonema (see later for additional support). In addition, self-synapsis of the X chromosome is associated with protection against elimination (Fig 1C).

To further confirm our findings that sex chromosome asynapsis drives oocyte elimination, we studied a second sex chromosome variant mouse model, the In(X)1H female. The In(X)1H mouse carries a large X chromosome inversion that disrupts X-X synapsis [27] and has previously been reported to experience prophase I oocyte loss [23]. While the majority of In(X)IH oocytes displayed normal X-X synapsis, defects in X-X synapsis were found in 15% of pachytene oocytes, and again we observed selection of these oocytes during diplonema (Fig 1D and 1F, S2D Fig).

Next, we investigated whether asynapsed autosomes also elicit oocyte elimination. We studied T(16;17)43H/+ (T43H/+) female mice [28], which carry an autosomal translocation involving chromosomes 16 and 17 and in which prophase I oocyte losses have not previously been reported. In T43H/+ females, 40% of pachytene oocytes had autosomal asynapsis (Fig 1G–1I). As with the XO and In(X)1H females, we observed selection against these oocytes as they progress through diplonema (Fig 1I and S2E Fig). Therefore, both sex chromosomal and autosomal asynapsis at pachynema lead to oocyte elimination during diplonema of prophase I.

Synaptic errors have previously been reported in wild type females [10,11]. We therefore examined whether a proportion of chromosomally wild type oocytes experience diplotene elimination. This was indeed the case: synaptic errors were found in 10% of XX oocytes at pachynema but in less than one percent at late diplonema (Fig 1J–1L, S2A and S2F Fig). We conclude that the meiotic surveillance mechanism operates in both chromosomally abnormal and wild type ovaries to eliminate oocytes with chromosomal asynapsis during diplonema (Fig 1M)

### DNA DSB markers do not persist on asynapsed chromosomes

**Fig 2:**
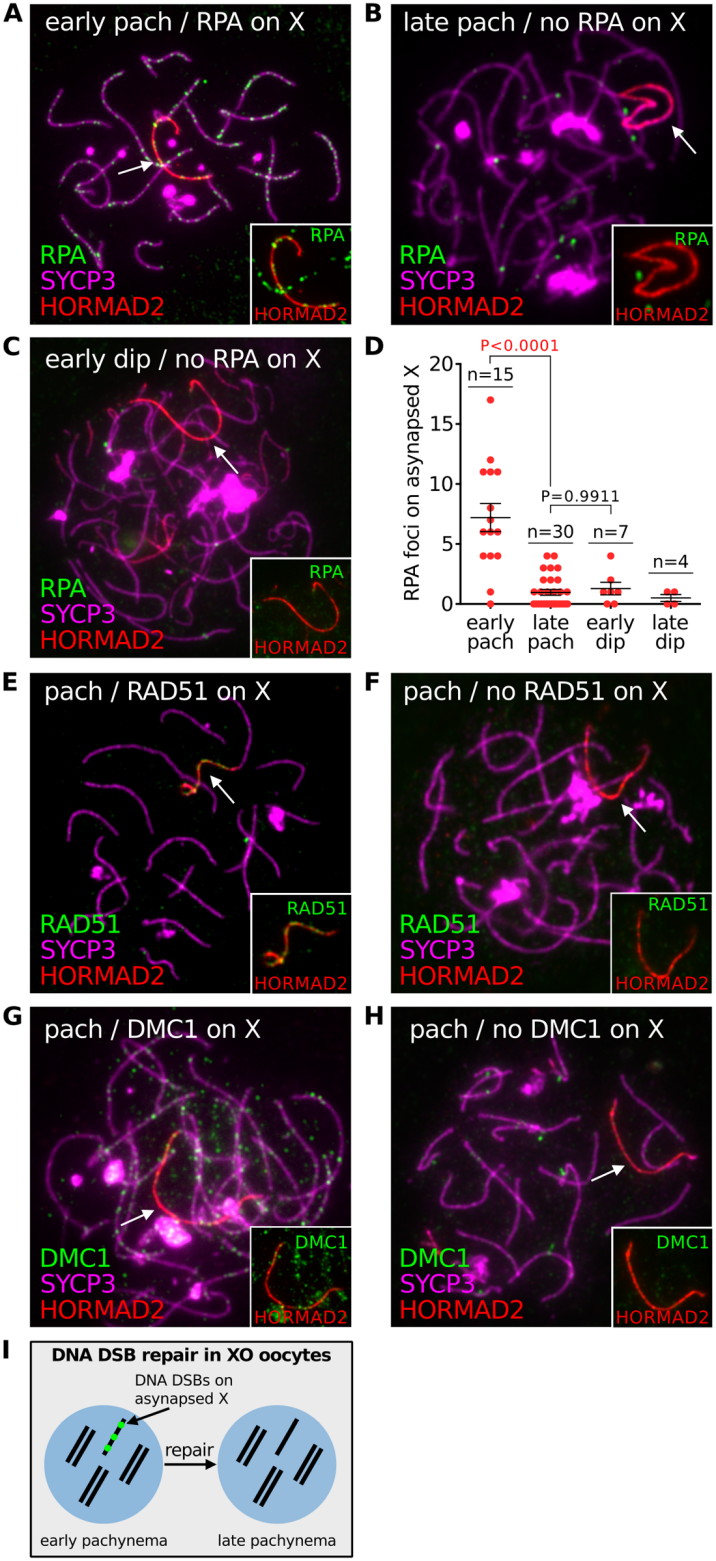
DNA DSB repair marker RPA does not persist on asynapsed chromosomes in XO female mice. (**A**) Early pachytene XO oocyte with numerous RPA foci on the asynapsed X chromosome, marked by HORMAD2 (arrow, inset). RPA foci are abundant on synapsed axes at early pachynema. (**B**) Late pachytene XO oocyte with no RPA foci on the asynapsed X chromosome (arrow, inset). RPA foci are less abundant on synapsed axes at late pachynema. (**C**) Early diplotene XO oocyte with no RPA foci on the asynapsed X chromosome (arrow, inset). (**D**) Number of RPA foci on the asynapsed X chromosome at early pachynema, late pachynema, early diplonema and late diplonema. (**E**) Pachytene XO oocyte with RAD51 foci on the asynapsed X chromosome (arrow, inset). (**F**) Pachytene XO oocyte with no RAD51 foci on asynapsed X chromosome (arrow, inset). (**G**) Pachytene XO oocyte with DMC1 foci on asynapsed X chromosome (arrow, inset). (**H**) Pachytene XO oocyte with no DMC1 foci on asynapsed X chromosome (arrow, inset). (**I**) Schematic showing turnover of DNA DSB repair protein (e.g. RPA) (red) on the asynapsed X chromosome by late pachynema.

Persistent meiotic DNA DSBs cause oocyte loss in targeted recombination mutants, e.g. *Dmc1* nulls [3]. Furthermore, a recent study has detected DNA damage foci in *Spo11 -/-* prophase oocytes [29], indicating that DNA DSBs may also contribute to elimination in this model. To understand if unrepaired DNA DSBs also have a role in the elimination of oocytes with chromosomal abnormalities, we determined whether DSBs persist on asynapsed chromosomes in chromosomally variant mice, using the XO female model. We first analyzed DSB repair on asynapsed chromosomes during pachynema and diplonema using triple immunostaining for SYCP3, HORMAD2 and the DNA damage marker RPA (Fig 2). At early pachynema, RPA foci were present on the asynapsed X chromosome in XO oocytes (Fig 2A and 2B). Importantly however, at late pachynema RPA counts were lower, with many oocytes exhibiting no RPA foci (Fig 2B and 2D). Thereafter, RPA counts remained low during early and late diplonema (Fig 2C and 2D). Analysis of DNA DSB turnover on the asynapsed X chromosome using other DNA damage markers, RAD51 and DMC1, gave similar results: no foci visible on the asynapsed X at late pachynema (Fig 2E–2H). This drop in DNA DSB repair protein counts cannot be explained by oocyte elimination, because no elimination occurs during pachynema (S2C Fig). We conclude that DNA DSBs are repaired on asynapsed chromosomes by the end of pachynema (Fig 2I). These findings mirror those in males, in which disappearance of RPA/RAD51/DMC1 foci on the asynapsed X chromosome occurs during pachynema [30] and is thought to reflect sister chromatid repair [31].

Notably, the behaviour of DNA DSB markers in chromosomally abnormal mice differed to that observed in *Dmc1 -/-* mutants. In this mutants, RPA foci on asynapsed chromosomes persisted during late prophase I (S3 Fig). These findings show that asynapsis-associated DNA DSBs behave differently in mice with and without targeted mutations of key meiotic genes.

### *H2afx* ablation increases oocyte numbers in XO females

**Fig 3:**
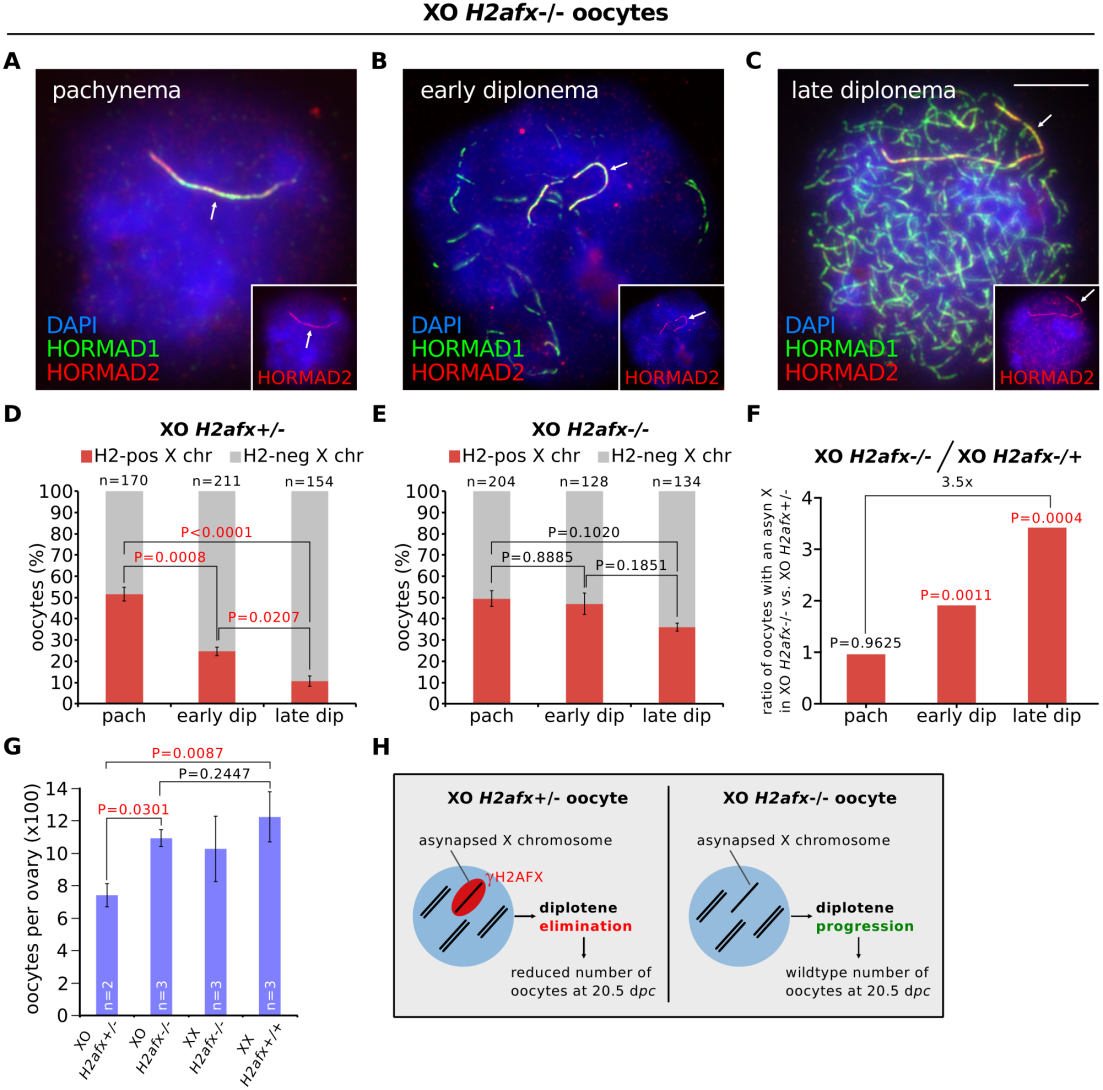
*H2afx* deletion reverses perinatal oocyte losses in XO females. (**A**) Pachytene XO *H2afx-/-* oocyte with an asynapsed X chromosome (arrow; marked by HORMAD1, green, and HORMAD2, red and inset). (**B**) Early diplotene XO *H2afx*-/- oocyte, showing intermediate levels of desynapsis (HORMAD1-only positive axes, green) and an asynapsed X chromosome (arrow; marked with both HORMAD1 and HORMAD2, inset). (**C**) Late diplotene XO *H2afx*-/- oocyte, showing extensive desynapsis and an asynapsed X chromosome (arrow). Scale bar represents 10μm. (**D**) Mean percentage (± s.e.m.) of XO *H2afx+/-* oocytes with a HORMAD1/2 double-positive X chromosome between pachynema and late diplonema at 19.5 d*pc*. Statistics were performed as in Fig 1. (**E**) Mean percentage (± s.e.m.) of XO *H2afx-/-* oocytes with a HORMAD1/2 double-positive X chromosome between pachynema and late diplonema at 19.5 d*pc*. No statistically significant difference was found between means at pachynema and late diplonema (Tukey’s test). (**F**) Enrichment of oocytes with an asynapsed X chromosome in XO *H2afx-/-* compared to XO *H2afx+/-* between pachynema and late diplonema. Enrichment was defined as the ratio of the mean percentage of oocytes with an asynapsed X in XO *H2afx-/-* versus XO *H2afx+/-* control, using data from part (D) and (E). (**G**) Mean number (± s.e.m.) of oocytes in XO and XX, ovaries with varying doses of *H2afx*, XO *H2afx-/-*,XX *H2afx+/-* and XX *H2afx+/+* at 20.5 d*pc*. n is the number of non-littermate ovaries analyzed. Tukey multiple comparison tests. (**H**) Summary showing the importance of H2AFX in XO oocyte elimination.

Curiously, while unrepaired DNA markers disappear from unsynapsed chromosomes during pachynema (Fig 2), γH2AFX is retained until the loss of asynaptic oocytes. Thus, our results indicate that oocyte elimination in chromosomally abnormal mice is linked to the presence of H2AFX serine-139 phosphorylation on the chromatin of asynapsed chromosomes. To verify this, we deleted *H2afx* in our XO mouse model system. We recently showed that *H2afx* is essential for meiotic silencing in females (Cloutier et al, submitted), as it is in males [32]. *H2afx* nullizygosity did not influence HORMAD1 and HORMAD2 localization to the asynapsed X chromosome (Fig 3A–3C) or the frequency of pachytene X self-synapsis (Fig 3D–3F) in XO females. Furthermore, the efficiency of autosomal synapsis (S4A Fig) and the number and timing of disappearance of RPA foci on the asynapsed X chromosome (S4B Fig) were unchanged in XO *H2afx-/-* mice relative to controls.

In our *H2afx* deletion experiments, we quantified the percentage of oocytes with an asynapsed X chromosome during successive prophase I substages using HORMAD1 and HORMAD2 double-immunostaining, which allowed us to distinguish the asynaptic X chromosome from desynapsing autosomes (Fig 3A–3C). HORMAD2 marks only asynaptic axis, while HORMAD1 marks both asynaptic and desynapsing axis [12]. In control XO *H2afx+/-* females, oocytes with an asynapsed X disappeared during diplonema (Fig 3D), as had been observed in XO *H2afx+/+* females (Fig 1C). Notably, however, in XO *H2afx-/-* females, there was no significant decrease in the percentage of oocytes with an asynapsed X chromosome during this period (Fig 3E). As a result, XO *H2afx-/-* females had 3.5 times as many late diplotene oocytes with an asynapsed X chromosome as control XO *H2afx+/-* siblings (Fig 3F). *H2afx* deletion had no effect on the percentage of oocytes with X chromosome asynapsis at pachynema (Fig 3D), further confirming that the prophase I elimination mechanism in chromosomally abnormal mice (Fig 1) operates during diplonema. Since serine-139 phosphorylation of H2AFX is the critical epigenetic event in silencing [33], we repeated this analysis using XO females carrying a non-phosphorylatable form of histone H2AFX mutated at serine-139 [34]. Importantly, these mice also exhibited retention of oocytes with an asynapsed X chromosome during diplonema, in contrast to control XO females (S4C Fig). We conclude that H2AFX phosphorylation is required for elimination of XO oocytes with asynapsis.

Next we confirmed the rescue in oocyte loss observed in XO *H2afx-/-* females by quantifying oocyte numbers in newborn mice. We compared the relative numbers of oocytes, as determined histologically, in XO *H2afx+/-* with XO *H2afx-/-* ovaries at 20.5 d*pc* (i.e. 1 day postpartum), when most oocytes have progressed to diplonema and when losses have previously been shown to occur in XO females [23]. Consistent with our previously observed rescue, we found that XO *H2afx-/-* females had significantly more oocytes than age-matched XO *H2afx+/-* females (Fig 3G). Furthermore, oocyte counts in XO *H2afx-/-* females were similar to those of XX *H2afx-/-* females (Fig 3G). Importantly, oocyte numbers were not significantly different between XX *H2afx-/-* and XX *H2afx+/+* females (Fig 3G), demonstrating that *H2afx* deletion has no effect on oocyte viability at this stage of oogenesis. In summary, H2AFX is required for the perinatal oocyte loss in XO females (Fig 3H).

### Distinguishing between the role of asynapsis and H2AFX-dependent silencing in oocyte loss

In mammals, asynapsis has been proposed to cause oocyte loss through a checkpoint mechanism responding to asynapsis or through H2AFX-associated silencing, but distinguishing between these models has proved challenging because putative synapsis checkpoint proteins are necessary for silencing [13,19–21]. The checkpoint model predicts that asynapsed chromosomes will cause elimination irrespective of their gene content. By contrast, under the silencing model, the outcome of asynapsis may be linked to the gene content of the asynapsed chromosome, e.g. whether it contains essential oogenesis genes. Cot1 RNA FISH analysis revealed high global gene expression levels in XX prophase I oocytes, especially during diplonema, indicating that all mouse chromosomes harbour oogenesis-expressed genes (S5A–S5D Fig). Therefore, to separate the effects of asynapsis and silencing, we used mice carrying additional chromosomes, so-called “accessory” chromosomes, which by definition carry non-essential genes. These accessory chromosomes are hemizygous and thus are asynapsed during meiosis. If asynapsis *per se* were the proximal trigger of oocyte elimination, then an accessory chromosome would cause oocyte losses. However, if meiotic silencing were responsible, then an accessory chromosome would not trigger elimination, because silencing would affect genes not normally present in the oocyte genome, and would therefore not be detrimental in this scenario.

**Fig 4:**
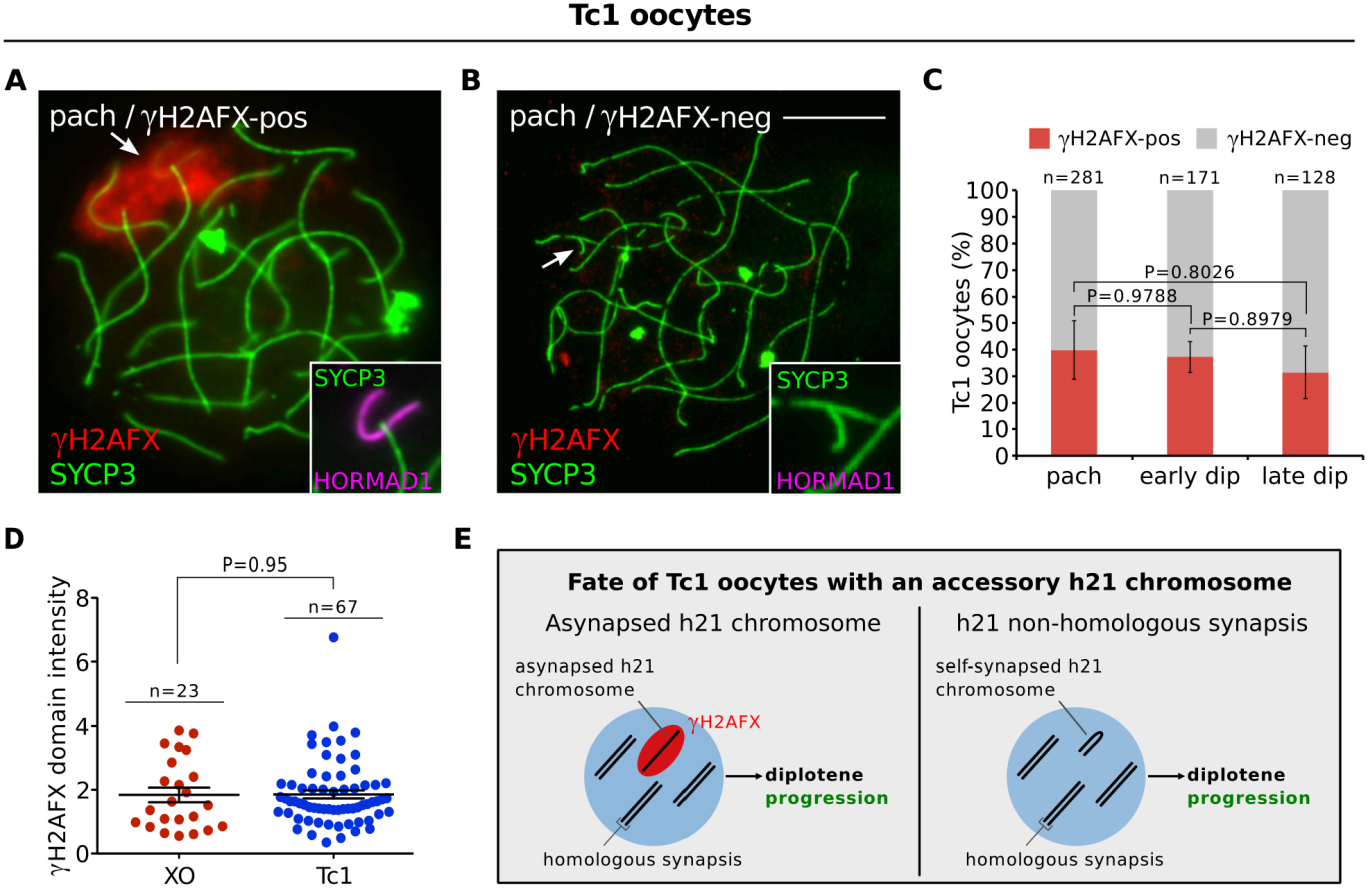
Oocytes in the Tc1 accessory chromosome mouse model do not exhibit diplotene elimination. (**A**) Pachytene Tc1 oocyte with an asynapsed h21 chromosome (arrow, inset), labelled with γH2AFX (red, arrow) and HORMAD1 (magenta, inset). (**B**) Pachytene Tc1 oocyte with self-synapsed h21 chromosome (arrow, inset), which is negative for γH2AFX and HORMAD1. Scale bar represents 10μm. (**C**) Mean percentage (± s.e.m.) of Tc1 oocytes with a γH2AFX-positive or γH2AFX-negative h21 chromosome between pachynema and late diplonema, demonstrating no significant elimination of Tc1 oocytes with asynapsed h21 chromosomes. n is the number of oocytes counted from three non-littermate mice at 18.5 d*pc*. (**D**) Comparison of γH2AFX domain integrated intensity between XO and Tc1 oocytes at diplonema, the stage when oocyte losses are observed in XO but not Tc1 females. n refers to the number of oocytes analyzed. (**E**) Schematic showing that the asynapsed h21 chromosome does not trigger oocyte losses at diplonema.

We first studied a Down syndrome mouse model, the Tc1 female, which carries a single copy of human chromosome 21 (h21). Asynapsed h21 chromosomes were present in 40% of pachytene oocytes (Fig 4A and 4C) and exhibited γH2AFX domains of size comparable to those in XO females (Fig 4D). In the remaining oocytes, the Tc1 chromosome had self-synapsed (Fig 4B and 4C). Remarkably, oocytes with h21 asynapsis were not eliminated during diplonema (Fig 4C and 4E, S5G Fig). This was in spite of the presence of HORMAD1 (Fig 3A) and other asynapsis-associated proteins on the h21 chromosome (S5E and S5F Fig). As observed in all previous chromosome variant models (Figs 1 and 2), RPA foci disappeared on the asynapsed TC1 chromosome during prophase I progression (S5H Fig).

To confirm our findings, we then studied a second supernumerary chromosome model, the sex-reversed XXY^d1^ female [35]. XXY^d1^ females harbor an accessory mouse Y chromosome, which by definition contains no oogenesis-essential genes. Notably, we also observed no selection against oocytes with an asynapsed Y^d1^ chromosome (S5I–S5K Fig). These data show that asynapsis *per se*, and the presence of asynapsis-associated proteins, are not sufficient to trigger oocyte elimination. In addition, they suggest that the oocyte loss in XO, In(X)1H, T43H and XX females (Fig 1) is instead linked to the gene content of the asynapsed chromosomes.

## Discussion

**Fig 5:**
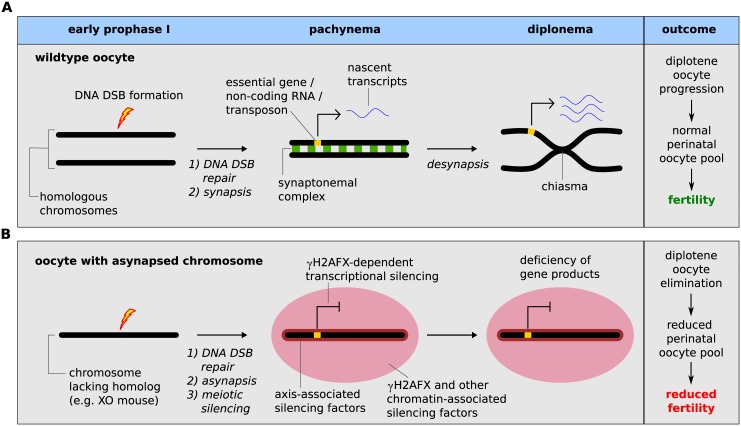
Meiotic silencing model of prophase I oocyte elimination. (**A**) Wild type oocyte: Meiotic DNA DSBs are formed during early prophase I by SPO11 (lightning bolt). During pachynema, homologous chromosomes synapse and meiotic DSB are repaired (DSB repair proteins shown as a red star). At diplonema, homologs desynapse and remain connected at crossover sites (chiasma). Essential genes (yellow block on chromosome) are actively transcribed during pachynema and diplonema (transcripts shown as blue ribbon). (**B**) Oocytes with a chromosome abnormality that disrupts meiotic pairing (i.e. XO oocyte): As in wild type oocytes, meiotic DNA DSBs are formed in early prophase I and are repaired on the asynapsed chromosome during pachynema. At pachynema, however, the chromosome lacks a homolog and remains asynapsed, triggering meiotic silencing, which is mediated both by axial-associated and chromatin-associated silencing factors, such as γH2AFX (red domain). This results in silencing of essential genes on the asynapsed chromosome. This consequence of essential gene silencing is oocyte elimination during diplonema and subsequent reduced fertility.

Here we have examined the mechanisms that cause prophase I oocyte loss in an extensive array of chromosomally variant mice, shedding new light on fundamental aspects of meiosis and on meiotic surveillance pathways (Fig 5). We show that oocyte loss in these models occurs during diplonema, later than predicted by the classical pachytene checkpoint model [17]. Furthermore, we find that DNA DSB foci disappear from asynapsed chromosomes during pachynema in chromosomally abnormal mice. DNA DSB repair was observed in all models studied, whether or not they exhibit prophase I oocyte elimination. Similar findings have been made in other models, e.g. *Sycp3* null mice, in which surviving oocytes were found to resolve axial element-associated DNA DSBs [36]. We attribute the disappearance of DNA DSB foci to the existence in the mammalian oocyte of a repair mechanism for asynapsis-associated DNA DSBs that occurs independent of *H2afx* (S4B Fig). This most likely proceeds via inter-sister repair, which occurs more commonly during meiosis than previously thought [37]. Importantly, the behaviour of asynapsis-associated DNA DSBs in chromosomally abnormal mice differs from that seen in recombination defective mice, e.g. *Dmc1-/-* mutants. This is presumably because the targeted genes are themselves necessary for DNA repair, and thus their absence causes persistent DNA damage [38]. This difference emphasises the importance of studying both targeted and non-targeted mouse models to gain a full understanding of the pathways causing prophase I oocyte elimination.

We also show that deletion of *H2afx* rescues perinatal oocyte loss in XO females. How H2AFX phosphorylation drives oocyte elimination is unclear. However, an important finding comes from our analysis of accessory chromosome mice: this shows that the presence of asynapsed chromosomes *per se* and of asynapsis-associated factors, e.g. HORMAD1, HORMAD2, BRCA1, ATR and γH2AFX, is insufficient to cause diplotene oocyte elimination. This observation cannot be readily explained by simple synapsis checkpoint models. While it is conceivable that accessory chromosomes somehow do not efficiently activate such a checkpoint, we find it unlikely, since our experiments reveal no qualitative or quantitative differences in the asynapsis response between these models and those that exhibit diplotene oocyte loss. We therefore favour a scenario in which the differential effect of γH2AFX on oocyte fate in chromosomally abnormal versus accessory chromosome mice is due to the gene content of the asynapsed chromosome.

H2AFX phosphorylation could cause elimination through silencing of germ cell-specific or housekeeping protein-coding, or non-coding genes, or indirectly through changes in the balance of transcription factor binding profiles on asynapsed versus synapsed chromosomes. Our data do not allow us to discriminate between these possibilities. However, when combining our observations in the female with those in the male germ line, the most parsimonious model currently invokes a role for silencing of germ-cell expressed genes in prophase I loss. During male meiosis, accumulation of HORMAD1, HORMAD2, BRCA1, ATR, MDC1, and γH2AFX at asynapsed autosomes causes prophase I elimination, but localization of the same proteins to the asynapsed X chromosome does not. The X chromosome does not possess unique properties preventing it from triggering loss. This is demonstrated by the fact that asynapsed accessory chromosomes, such as in Tc1 males, also fail to trigger prophase I elimination (S6A–S6C Fig). Interestingly, however, in contrast to the autosomes, the X chromosome is dramatically depleted in genes required for male meiosis [39,40]. Furthermore, silencing of X-linked housekeeping genes, e.g. *Pgk1*, *Gdpdx* is compensated for by a unique system of autosomally-located, X-derived retrogenes that are expressed only in males and are essential for spermatogenesis [39,41,42]. The fact that both the X chromosome and accessory chromosomes are deficient in male meiotic genes could readily explain why H2AFX-induced silencing of these chromosomes does not induce prophase I loss.

Our analysis shows that oocyte elimination occurs during diplonema, i.e. perinatally, in all models studied, irrespective of the identity of the asynapsed chromosome. Perinatal oocyte loss is also observed in other models exhibiting asynapsis and associated meiotic silencing, e.g. *Spo11* null females. Under the meiotic silencing model, the prediction would be that all chromosomes house genes required either specifically for late prophase I, or for general housekeeping functions. We note that the asynapsed chromosomes studied in our model systems carry such genes: aside from aforementioned housekeeping genes, the X chromosome is enriched in genes required for oogenesis [43], including *Zfx* [44], *Bmp15* [45] and *Fmr1* [46]. The region of maximum chromosome 17 asynapsis in T43H/+ females [47] also contains critical genes, including the splicing factor *Srpk1*, ribosome *Rpl10a* and mRNA capping factor *Cmtr1*. Given that meiotic silencing covers megabase-scale chromosome regions, inactivating hundreds of genes, oocyte loss could result from the contemporaneous disturbance of multiple biological pathways. How such disturbances trigger oocyte demise is unclear. Although we observe TUNEL-positive oocytes in XO females at 19.5d*pc*, when asynapsis-associated oocyte elimination is taking place, the numbers are not significantly elevated relative to XX females (S7 Fig).

Finally, a previous study examined the effect of ablating meiotic silencing in *Smc1b* null oocytes, and concluded that silencing had a limited role in oocyte loss [10]. In those experiments, meiotic silencing was prevented by removing the axial element component *Sycp3*. While some degree of oocyte rescue was observed perinatally, the effect was only transient, and was not sustained at 4d*pp*. However, it should be noted that *Smc1b* null oocytes harbour additional lesions, e.g. defective recombination, that are not present in the chromosomally abnormal mouse models studied herein, and could independently trigger oocyte elimination [48]. Thus, in targeted mutants, multiple surveillance pathways could conspire to drive oocyte elimination.

## Materials and Methods

### Ethics statement

All animal procedures were in accordance with the United Kingdom Animal Scientific Procedures Act 1986 and were subject to local ethical review.

### Animals

Females were set up in matings and checked daily for copulation plugs. The day of plugging was considered 0.5 days *post coitum* (d*pc*). Embryos were sacrificed at 17.5, 18.5, 19.5 and 20.5 d*pc* using UK Home Office Schedule I methods. Ovaries were dissected from embryos and flash frozen in liquid nitrogen. Material was stored at -80°C until later use. All mice were maintained according to UK Home Office regulations. XO mice were generated on a random bred MF1 background (NIMR stock) by mating XX females to fertile X^Y^*O males, which harbor an X chromosome fused with a Y chromosome and give rise to ‘O’ gametes [49]. T(16;17)43H mice were maintained on a C57BL/10ScSnPh (B10) background, as previously described [28,47]. *H2afx-/-* mice [50] were generated on the MF1 background. XO *H2afx-/-* mice were generated by crossing X^Y^O *H2afx+/-* males with XX *H2afx+/-* females. *Dmc1-/-* mice [38] were produced on a C57BL/6 background. Tc1 mice [51] were maintained on the MF1 background. The C57BL/6 males used in this cross were maintained on-site at NIMR. XXY ^d1^ females were produced on an MF1 background by mating XY males to sex-reversed XY^d1^ females [35]. Mice carrying the *H2afx* ^S136/139A^ transgene were described previously [52].

### Chromosome spreads

Surface spreads and chromosome painting were performed as previously described [16,53]. The following primary antibodies were used for immunofluorescent experiments: rabbit anti-SYCP3 (1:100, Abcam: ab15093), mouse anti-γH2AFX (1:100, Upstate: 16–193; 1:100), guinea pig and rabbit anti-HORMAD1 and anti-HORMAD2 (ref. [12], 1:200), rabbit anti-RPA32/RPA2 (Abcam, ab-10359; 1:100), rabbit anti-BRCA1 (1:100, gift from Chu-Xia Deng), goat anti-ATR (1:50, Santa Cruz: sc-1887). Primary antibody incubations were carried out overnight at 37°C and secondary antibody incubations for 1hr at 37°C. For chromosome spread analyses, oocytes were first categorized into meiotic substages based upon SYCP3 and HORMAD1 staining, as described in S1 Fig. After the meiotic substage was determined, the oocytes were then assessed for γH2AFX domains or HORMAD2 axial staining (for XO *H2afx*-/- experiments). For quantification of the γH2AFX signal in spread oocytes (Fig 4D), images were taken with matched exposure times. We calculated the integrated intensity of γH2AFX domain area using Fiji software [54]. For each image we estimated background intensity in a region outside of the nucleus. We then calculated the background-adjusted intensity value of the γH2AFX domains by subtracting the background equal to the area of the γH2AFX domain.

### Oocyte counting

Ovaries were harvested from females at 20.5 days *post-coitum* (d*pc*), fixed in 4% paraformaldehyde overnight at 4°C and then transferred to 70% ethanol. Fixed ovaries were dehydrated by three successive 5min incubations with 95% ethanol, 100% ethanol, 100% xylene and were then embedded in paraffin wax. Ovaries were serially sectioned at 5–7μm thickness. Sections were dewaxed using histoclear (2x5min) and 1:1 histoclear:ethanol (1x5min), and then rehydrated using the following ethanol series: 100% ethanol (2x5min), 95% ethanol (1x5min), 80% (1x5min), 70% (1x5min), 50% (1x3min), and PBS (1x5min). Sections were stained with DAPI and oocytes were identified based upon their distinct size and nuclear cytology, as described previously [23]. To quantify the relative number of oocytes in each ovary, we summed the oocyte counts from every tenth section, as described previously [19].

### TUNEL analysis

Tunel analysis was performed using the ApopTag Plus Peroxidase *In Situ*, Apoptosis Detection Kit, S710, Millipore, according to manufacturer’s instructions.

### Statistical analyses

Statistical analysis was performed using GraphPad Prism 6.0. For statistical comparison of means, a Tukey-Kramer multiple comparison test (for >1 comparisons) or an unpaired t test (for one comparison) was performed. Calculated P values are reported in figures or legends.

### Imaging

Imaging was performed using an Olympus IX70 inverted microscope with a 100-W mercury arc lamp. For chromosome spread and RNA FISH imaging, an Olympus UPlanApo 100x/1.35 NA oil immersion objective was used. For ovary section imaging, an Olympus UPlanApo 20x/0.75 NA objective was used. A Deltavision RT computer-assisted Photometrics CoolsnapHQ CCD camera with an ICX285 Progressive scan CCD image sensor was utilized for image capture. 16-bit (1024x1024 pixels) raw images of each channel were captured and later processed using Fiji. Quantitation of Cot1 and γH2AFX intensities was performed as previously described [55].

## Supporting Information

S1 Fig Oocyte substaging criteria. For all chromosome spread analyses, oocytes were categorized into the substages pachynema, early diplonema and late diplonema based upon the staining pattern of SYCP3 and HORMAD1. (**A**) XX pachytene oocyte. At pachynema, SYCP3 labels 20 fully synapsed chromosome pairs. HORMAD1, if present at all, stains very weakly. (**B**) XX early diplotene oocyte. At early diplonema, homologous chromosomes show intermediate levels of desynapsis, marked by HORMAD1 axial accumulation. At this stage, segments of synapsis are still present, shown by SYCP3-positive and HORMAD1-negative axial segments. (**C**) XX late diplotene oocytes. At late diplonema, all chromosome pairs have desynapsed, as evident by complete HORMAD1/SYCP3 co-localization. At this stage SYCP3 and HORMAD1 also show signs of fragmentation. Scale bar represents 10μm.
(EPS)

S2 Fig Oocytes with asynapsed chromosomes are eliminated during diplonema, as determined by analyses of different gestational ages. (**A**) The mean percentage (± s.e.m.) of XX oocytes at pachynema, early diplonema and late diplonema at 17.5 d*pc*, 18.5 d*pc* and 19.5 d*pc*. During this period of gestation, oocytes progress in a semi-synchronous wave from pachynema to late diplonema. Three non-littermate ovaries were analyzed per age, and 100–200 oocytes were counted per ovary. (**B**) The mean percentage (± s.e.m.) of XO oocytes with a γH2AFX domain at 17.5 d*pc*, 18.5 d*pc*, and 19.5 d*pc*. (**C**) The mean percentage (± s.e.m.) of XO oocytes with a γH2AFX domain at early and late pachynema, using RPA substaging criteria. **(D-F**) Mean percentage (± s.e.m.) of oocytes with a γH2AFX domain at 17.5 d*pc*, 18.5 d*pc* and 19.5 d*pc* in (**D**) In(X)1H females, (**E**) T43H females, and (**F**) XX females. n is to the number of non-littermate ovaries analyzed per gestational age. 100–200 oocytes were counted per ovary. Tukey multiple comparison tests.
(EPS)

S3 Fig *Dmc1*-/- oocytes have persistent RPA foci. Three *Dmc1*-/- oocytes from ovaries harvested at 19.5 d*pc*, when meiosis has reached the diplotene stage. Each oocyte has extensive asynapsis, as shown by SYCP3 (blue) and HORMAD2 (red) co-localization, and persistent RPA foci (green). This pattern was observed in all of n = 50 diplotene oocytes.
(EPS)

S4 Fig Meiotic characterization of XO *H2afx*-/- oocytes. (**A**) Mean percentage (± s.e.m.) of XO *H2afx*+/- and XO *H2afx-/-* oocytes with multiple asynapsed chromosomes, indicative of autosomal asynapsis (red bar). Blue bars represent the percentage of oocytes showing no autosomal asynapsis. There are no significant differences between the means, indicating that autosomal synapsis is unaffected by *H2afx* deletion. n is the number of oocytes analyzed. (**B**) Number of RPA foci on the asynapsed X chromosome at pachynema, early diplonema, and late diplonema in XO *H2afx*+/+ and XO *H2afx-/-* oocytes. n is the number of oocytes analyzed. Tukey’s test was used to determine the significance and n.s. means not significant. (**C**) The mean percentage (± s.e.m.) of oocytes with an asynapsed X chromosome at pachynema and diplonema in XO *H2afx-/- H2afx* ^S139A^ females and XO *H2afx+/- H2afx* ^S139A^ controls. No diplotene elimination occurs in XO *H2afx*-/- oocytes carrying a non-phosphorylatable *H2afx* ^S139A^ transgene. This transgene encodes a non-phosphorylated version of H2AFX at serine 139 (S139A), as described previously [34]. n is the number of ovaries analyzed per genotype, and at least 100 oocytes were counted per ovary. Tukey multiple comparison tests.
(EPS)

S5 Fig Cot1 transcription analysis in XX oocytes and lack of diplotene elimination in oocytes with accessory chromosomes. (**A-D**) Cot-1 RNA FISH (green) analysis of global transcription in XX oocytes. Representative images of XX oocytes at (**A**) pachynema, (**B**) early diplonema, and (**C**) late diplonema, as determined by HORMAD1 staining (red, inset). Asterisks in panel (**C**) represents Cot-1 “holes” that coincide with sites of constitutive heterochromatin. (**D**) Quantification of nuclear Cot-1 RNA staining intensity. n refers to the number of cells analyzed for each cell type. Tukey multiple comparison test. (**E-H**) Tc1 oocytes with a single copy of human chromosome 21 (h21). The asynapsed h21 is enriched in asynapsis-associated proteins, including (**E**) BRCA1 and (**F**) ATR. (**G**) Mean percentage (± s.e.m.) of Tc1 oocytes with an asynapsed (γH2AFX-positive) h21 chromosome (red bar) at 17.5 d*pc*, 18.5 d*pc* and 19.5 d*pc*, demonstrating that oocytes with an asynapsed h21 are not eliminated during this period. n is the number of ovaries analyzed, and at least 100 oocytes were counted per ovary. Tukey multiple comparison test. (**H**) Number of RPA foci on the asynapsed h21 chromosome. Tukey multiple comparison test. (**I-K**) XXY^d1^ oocytes with single copy of the mouse Y^d1^ chromosome. (**I**) XXY^d1^ pachytene oocyte with an asynapsed (γH2AFX-positive, red) Y^d1^ chromosome (arrow). Inset shows accumulation of HORMAD1 (magenta) along asynapsed Y^d1^ chromosome. (**J**) XXY^d1^ oocyte with self-synapsed Y^d1^ chromosome (arrow). Scale bar represents 10μm. (**K**) Percentage of XXY^d1^ oocytes with a γH2AFX domain (red bar) between pachynema and late diplonema, demonstrating that oocytes with an asynapsed Y^d1^ are not eliminated during diplonema. n is the number of oocytes analyzed.
(EPS)

S6 Fig Histological comparison of Tc1 and wildtype male seminiferous tubules. (**A**) Tc1 male testis section. Arrow shows a stage XII tubule with a clear MI block, resulting in MI pile-up and a dearth of post-meiotic stages. Some cells still progress giving rise to spermatids. Arrowhead shows a stage XI tubule with normal diplotene cell progression, demonstrating that Tc1 spermatocytes are not eliminated in diplonema. (**B**) Wildtype testis section showing a normal stage XII tubule (arrow) with no MI block and normal post-meiotic stages. (**C**) WT testis section showing a normal stage XI tubule (arrowhead).
(EPS)

S7 Fig TUNEL analysis in XO and XX females at 19.5d*pc*. **(A)** Section through XO ovary showing TUNEL-positive, i.e. apoptotic oocytes. **(B)** Section through XX ovary showing TUNEL-positive, i.e. apoptotic oocytes. **(C)** Quantitation of TUNEL analysis (n = 3 females for each genotype). Although the number of TUNEL positive oocytes is elevated in the XO relative to the XX female, this elevation is not statistically significant.
(EPS)
