# Correction: Histone H2AFX Links Meiotic Chromosome Asynapsis to Prophase I Oocyte Loss in Mammals

**DOI:** 10.1371/journal.pgen.1005753

**Published:** 2015-12-15

**Authors:** The PLOS Genetics Staff

Fig 1 is incorrect, as it is missing Part M. A corrected version is here. The publisher apologizes for the error.

**Fig 1:**
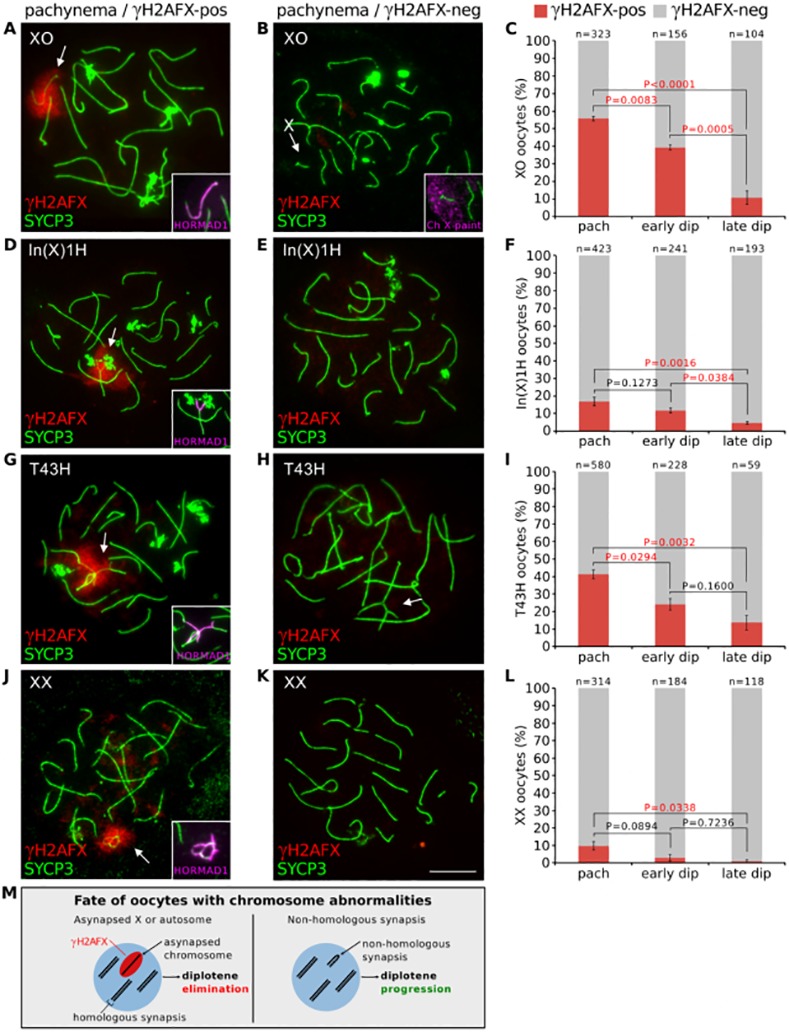
Oocytes with asynapsed chromosomes are eliminated during diplonema. (**A**) XO pachytene oocyte with asynapsed X chromosome (arrow). SYCP3 (green) marks chromosome axes, γH2AFX (red) marks chromatin associated with asynapsed axes, and HORMAD1 (magenta, insets) marks asynapsed axes. (**B**) XO pachytene oocyte with self-synapsed X chromosome. The self-synapsed X chromosome (arrow) was identified by X chromosome painting (magenta, inset). (**C**) The mean percentage (± s.e.m.) of XO oocytes with a γH2AFX-positive or γH2AFX-negative X chromosome at pachynema, early diplonema and late diplonema. (**D**) In(X)1H pachytene oocyte with asynapsis (arrow). (**E**) In(X)1H pachytene oocyte with fully synapsed chromosomes. (**F**) Mean percentage of In(X)1H oocytes with γH2AFX-positive or γH2AFX-negative X chromosomes at pachynema, early diplonema and late diplonema. (**G**) T43H pachytene oocyte with asynapsed autosomes (arrow). (**H**) T43H pachytene oocyte with fully synapsed chromosomes, showing a trivalent involving the translocated chromosomes (arrow). (**I**) Mean percentage of T43H oocytes with γH2AFX-positive or γH2AFX-negative autosomes at pachynema, early diplonema and late diplonema. (**J**) XX pachytene oocyte with asynapsis (arrow). (**K**) XX pachytene oocyte with fully synapsed chromosomes. (**L**) Mean percentage of XX oocytes with γH2AFX-positive or γH2AFX-negative chromosomes at pachynema, early diplonema and late diplonema. P values were calculated from Tukey multiple comparison tests. Significant P values (P<0.05) are denoted by red font. Scale bar represents 10μm. (**M**) Schematic illustrating the possible outcomes for oocytes with chromosome abnormalities.
